# MALDI-TOF mass spectrometry: a new tool for rapid identification of cercariae (Trematoda, Digenea)

**DOI:** 10.1051/parasite/2019011

**Published:** 2019-03-06

**Authors:** Antoine Huguenin, Jérôme Depaquit, Isabelle Villena, Hubert Ferté

**Affiliations:** 1 EA 7510, ESCAPE, Laboratoire de Parasitologie-Mycologie, Université de Reims Champagne-Ardenne 51 rue Cognacq Jay 51092 Reims CEDEX France; 2 Laboratoire de Parasitologie Mycologie, CHU de Reims, Hôpital Maison Blanche 45 rue Cognacq Jay 51092 Reims CEDEX France; 3 USC ANSES Transmission vectorielle et épidémiosurveillance de maladies parasitaires (VECPAR) Reims France

**Keywords:** MALDI-TOF, Trematoda, Furcocercariae, identification, High-throughput identification, snails

## Abstract

Identification of cercariae was long based on morphological and morphometric features, but these approaches remain difficult to implement and require skills that have now become rare. Molecular tools have become the reference even though they remain relatively time-consuming and expensive. We propose a new approach for the identification of cercariae using MALDI-TOF mass spectrometry. Snails of different genera (*Radix*, *Lymnaea*, *Stagnicola*, *Planorbis*, and *Anisus*) were collected in the field to perform emitting tests in the laboratory. The cercariae they emitted (*Trichobilharzia anseri*, *Diplostomum pseudospathaceum*, *Alaria alata*, *Echinostoma revolutum*, *Petasiger phalacrocoracis*, *Tylodelphys* sp., *Australapatemon* sp., *Cotylurus* sp., *Posthodiplostomum* sp., *Parastrigea* sp., *Echinoparyphium* sp. and *Plagiorchis* sp.) were characterized by sequencing the D2, ITS2 and ITS1 domains of rDNA, and by amplification using specific *Alaria alata* primers. A sample of each specimen, either fresh or stored in ethanol, was subjected to a simple preparation protocol for MALDI-TOF analysis. The main spectral profiles were analyzed by Hierarchical Clustering Analysis. Likewise, the haplotypes were analyzed using the maximum likelihood method. Analytical performance and the log-score value (LSV) cut-off for species identification were then assessed by blind testing. The clusters obtained by both techniques were congruent, allowing identification at a species level. MALDI-TOF enables identification at an LSV cut-off of 1.7 without false-positives; however, it requires more data on closely related species. The development of a “high throughput” identification system for all types of cercariae would be of considerable interest in epidemiological surveys of trematode infections.

## Abstract (fr)

L’identification des cercaires a longtemps été basée sur des caractéristiques morphologiques et morphométriques, mais ces approches restent difficiles à utiliser et nécessitent des compétences devenues rares de nos jours. Les outils moléculaires sont devenus la référence mais restent relativement coûteux en temps et en argent. Nous proposons une nouvelle approche pour l’identification des cercaires à l’aide de la spectrométrie de masse MALDI-TOF. Des mollusques de différents genres (*Radix*, *Lymnaea*, *Stagnicola*, *Planorbis*, *Anisus*) récoltés en milieu naturel ont été soumis à des tests d’émission au laboratoire. Les cercaires émises (*Trichobilharzia anseri*, *Diplostomum pseudospathaceum*, *Alaria alata*, *Echinostoma revolutum*, *Petasiger phalacrocoracis*, *Tylodelphys* sp., *Australapatemon* sp., *Cotylurus* sp., *Posthodiplostomum* sp., *Parastrigea* sp., *Echinoparyphium* sp. et *Plagiorchis* sp.) ont été caractérisées par séquençage des domaines D2, ITS2 et ITS1 de l’ADNr et par l’amplification d’amorces spécifiques d’*Alaria alata*. Un échantillon de chaque échantillon, frais ou stocké dans l’éthanol, a été soumis à un protocole de préparation simple pour l’analyse MALDI-TOF. Les spectres de référence obtenus (Main Spectra Profiles : MSP) ont été analysés par HCA (Hierarchical Clustering Analysis). Parallèlement, les haplotypes ont été analysés en Maximum de vraisemblance. La performance analytique et le seuil des valeurs de log-score (LSV) pour l’identification des espèces ont ensuite été évalués par des tests à l’aveugle. Les groupes obtenus par les deux techniques étaient congruents, permettant une identification à un niveau spécifique. Une valeur seuil de LSV à 1,7 a permis l’identification de cercaires en MALDI-TOF sans faux-positif, mais davantage de données sur les espèces étroitement apparentées sont nécessaires pour valider ce seuil. La mise au point d’un système d’identification “à haut débit” pour tous les types de cercaires présenterait un intérêt majeur pour les enquêtes épidémiologiques sur les trématodoses.

## Introduction

In the life cycle of Trematoda, the first intermediate host is a mollusc, usually an aquatic snail. This host releases cercariae into the environment. Cercariae are free living mobile larval stages that must locate a suitable second definitive or intermediate host [17, 33]. The study of cercariae is therefore essential to understand the epidemiology of Trematoda and their ecological relationships with their hosts. Trematodes are one of the most important parasites in medical and veterinary parasitology. For example, the furcocercariae of *Schistosoma* are the causal agent of schistosomiasis which affects more than 230 million people worldwide [6], and the *Fasciola hepatica* fluke is a parasite of high importance in veterinary medicine [26].

Traditional identification of cercariae is based on their natural environment (fresh or saline water), the species of emitting molluscs, and their morphological features (presence of eye spots, type of tail, position of suckers, osmotic regulation system, and distribution of sensory papilla). However, this approach presents several limitations. The morphology of different species within the same genus is very similar at the cercarial stage, which renders species identification particularly challenging. The required expertise for identification is long to acquire and it is the prerogative of a small number of specialists whose number is in constant decline. Particular technical skills are also required for several coloration techniques such as silver-impregnation and borax-carmine staining [7, 11].

Molecular biology is becoming the gold standard for the identification of Trematodes at larval stages. Use of the D2 domain of the 28S subunit and the internal transcribed spacers (ITS2 and ITS1) of ribosomal DNA (rDNA), or the cytochrome C oxidase I (COI) gene of mitochondrial DNA has made it possible to refine the taxonomy of Trematodes [2]. Molecular techniques enable researchers to differentiate cryptic species that are morphologically similar at the larval or adult stages [14, 20]. These molecular tools have great discriminatory power, but (i) they are still sometimes technically challenging and remain time- and resource-consuming, and (ii) GenBank does not include sufficient sequences to allow for strong species identification, especially sequences obtained from adults (except for the most common parasites of human and veterinary importance).

Matrix-Assisted Laser Desorption/Ionization Time-Of-Flight Mass Spectrometry (MALDI-TOF MS) is now a widely used technique for easy, rapid, and reliable routine identification of bacteria and yeasts [5, 31, 34]. This technique is based on laser ionization of sample proteins after co-crystallization with MALDI-matrix and comparison of the obtained mass spectra with a database of reference spectra [5]. MALDI-TOF MS is currently under development for the study of protozoa with potential use for the identification of *Leishmania* [22], *Plasmodium* [23] and trypanosomatids [1]. Few applications, however, have been proposed in the field of helminthology. MALDI-TOF has recently proved its effectiveness for the rapid identification of *Trichinella* at the genus and species levels, with a high degree of confidence [27]. In the case of Trematodes, MALDI-TOF MS has been used to find biomarkers for schistosomiasis in mice sera, allowing for very early detection of the infection in this animal model [18].

We propose the use of MALDI-TOF MS as a rapid and inexpensive method for high-throughput identification of cercariae.

The goal of the present study was to design a simple protocol for acquiring MALDI-TOF spectra of cercariae freshly emitted from snails. The discriminatory power of this technique was then investigated and formed a preliminary spectral database especially targeting the furcocercariae of diplostomoids. The analytical performance of this technique was also evaluated by performing blind validation. Finally, we studied the effect of storage in ethanol on cercariae identification.

## Materials and methods

### Cercaria and snail collection

Snails from four different areas were collected: the first one, regularly prospected during an epidemiological survey of the transmission of *Alaria alata*, is located in the center of France [(National Domain of Chambord (DNC): 48°35′N 1°55′E)]; the second is Der-Chantecoq lake (DR) in North-Eastern France (48°35′N 4°45′E), the third was investigated in the context of human cercarial dermatitis from a recreational pond used for swimming [Zebulle Park/Chevenon (ZE) (46°91′N 3°22′E)], and the fourth is in a landscape of meadows in the locality of Jouaignes (JO) (49°30′N 3°53′E).

Snails were collected by hand from April 2017 to June 2018. They were collected once in all areas, except for the DNC area where the collection was performed monthly from spring to summer.

Collections were pooled in the laboratory and cercarial emergence was stimulated by lighting for 30 min to 2 h. Snails from positive batches were individualized for a second assay and preliminary screening of cercariae was performed using morphological features as proposed by Combes et al. [7] and Faltýnková et al. [10, 11]. Identification of snails was performed at the genus level according to Glöer and Meier-Brook [15]. Taking into account the fact that snails usually emitted one kind of cercariae, and after checking under a stereomicroscope, some of the cercariae were processed for MALDI-TOF, whereas others were preserved in 95% ethanol for molecular analysis. Some samples from the foot of most positive snails were also collected. DNA extraction was performed using a QIAamp DNA mini kit (Qiagen, Germany), following the manufacturer’s instructions.

In order to achieve molecular identification of the cercariae, we used the primers designed by Mollaret et al. [28] to amplify (i) the D2 domain of rDNA: C2’b (5′-GAAAAGTACTTTGRARAGAGA-3′) and D2 (5′-TCCGTGTTTCAAGACGGG-3′); and those previously used by Dvorák et al. [9], to amplify (ii) the second internal transcribed spacer ITS2 (ITS3Trem 5′-GCG TCG ATG AAG AGT GCA GC-3′ and ITS4Trem 5′-TCC TCC GCT TAT TGA TAT GC-3′), and (iii) the ITS1 (ITS2Trem, 5′-GCT GCA CTC TTC ATC GAC GC-3′ and ITS5Trem, 5′-GGA AGT AAA AGT CGT AAC AAG G-3′). Furthermore, specific *Alaria alata* primers [ALAITS1 (5′-GGC TTG GGA GTA GGT TCC TG-3′) and ALAITS2b (5′-GGT ATG TGT TGG CTG CTA GA-3′)] were used to perform rapid identification of all cercariae exhibiting forked tails (LF/FO) according to Portier et al. [32].

Two snail’s mitochondrial markers were amplified with the primers and in the conditions given by Jørgensen et al. [19]: large subunit 16S with 16Sar-L/16Sbr-H (5′-CGC CTG TTT ATC AAA AAC AT-3′/5′-CCG GTC TGA ACT CAG ATC ACGT-3′), and COI with ASMIT1/ASMIT2 (5′-TTT TTG GGC ATC CTG AGG TTT AT- 3′/5′-TAA AGA AAG AAC ATA ATG AAA ATG-3′). PCR products were directly sequenced in both directions with the primers used for DNA amplification (Genoscreen, France). Sequence alignments were performed by ClustalW in BioEdit [16].

Sequence homology was evaluated by nucleotide BLAST requests (https://blast.ncbi.nlm.nih.gov/Blast.cgi). A lack of homology was considered for values lower than 97%.

The evolutionary history was inferred by using the maximum likelihood method. The best evolution model (General Time Reversible model; GTR) with invariant sites was selected based on Akaike’s Information Criterion (AIC) and Bayesian Information Criterion (BIC) using MEGA7 built-in function [21].

Initial tree(s) for the heuristic search were obtained automatically by applying the Neighbor-Join and BioNJ algorithms to a matrix of pairwise distances estimated using the Maximum Composite Likelihood (MCL) approach, and then selecting the topology with superior log likelihood value. Internal node support was assessed by a bootstrap test over 500 replicates. All positions containing gaps and missing data were eliminated. All evolutionary analyses were conducted in MEGA7.

### MALDI-TOF MS Spectral acquisition

To achieve MALDI-TOF spectral acquisition, 2–5 μL of water containing freshly emerged cercariae was directly spotted to the MALDI target or centrifuged at 4000 rpm for 3 min. After centrifugation, the pellet was washed with distilled water and 5 μL was spotted onto the MALDI target plate (Bruker Daltonik GmbH, Bremen, Germany). Each sample was deposited in at least four replicates. After drying at room temperature, the samples were covered with 1 μL of 70% formic acid. After complete drying, 1 μL of matrix (α-cyano-hydroxy-cinnamic acid in solution with 2.5% trifluoroacetic acid and 50% acetonitrile in water, Bruker Daltonik) was added to each spot. The target was then air-dried at room temperature. MALDI-TOF spectrum acquisition was performed using a Microflex LT mass spectrometer controlled by FlexControl software (Bruker Daltonik) with detection of positive ions on a range of 2000–20,000 m/z (mass to charge ratio). Each spectrum was acquired from 240 laser shots on random regions of the spot using auto-execute mode. Instrument calibration was verified using the Bacterial Test Standard (Bruker Daltonik). Spectra were processed using the FlexAnalysis and MALDI-Biotyper v3.4 software suite (Bruker Daltonik). High quality spectra for each sample were selected to create reference spectra (Main Spectrum Profile: MSP) using the default Bruker Method, which were added to the in-house database. Hierarchical cluster analysis (MSP dendrogram) was performed on the newly created MSP using MALDI-Biotyper Compass Explorer v4.1 software, and a distance matrix was calculated using the correlation method and clustered with the Ward algorithm.

### Database validation and LSV cut-off determination

The newly created MSP database was evaluated by means of a blind test performed with new specimens from the DR lake. These new specimens were also deposited in four replicates and each spot was acquired 12 times. The log-score value (LSV) calculated by the Bruker MALDI-Biotyper was then used to evaluate the reliability of species identification based on the similarity between the reference MSP and newly acquired spectra. The cut-off for LSV was determined on the basis of molecular identification using a receptor-operated-channel curve (ROC curve) calculated by logistic regression (SAS 9.4, Grégy-sur-Yerres, France).

### Evaluation of the effect of storage in ethanol

In a first step, the specimens of the validation set stored in 80% ethanol were re-analyzed by MALDI-TOF 3 months (91 days) later, using the same parameters.

In a second step, specimens preserved in ethanol over a period ranging from 1 to 14 months were analyzed by MALDI-TOF. In order to evaluate the effect of ethanol fixation, some specimens were fixed in ethanol immediately after emission and analyzed by MALDI-TOF MS the same day. Differences between true positive rates and LSVs were analyzed using Chi-Square and ANOVA tests (SAS 9.4, Grégy-sur-Yerres, France).

## Results

A total of 2786 snails were tested for cercarial emission and only a few of them, belonging to the Lymnaeidae (*Radix*, *Lymnaea* and *Stagnicola*), and Planorbidae (*Planorbis* and *Anisus*) were positive. The number of the snails tested for each site and the labels of samples used for analysis are reported in the Table 1.

**Table 1:** Geographic origin of snails.

Site	Identification of snails	Number analysed	Prevalence (%)	Positive snails	Reference of positive snails
Der-Chantecocq lake (DR)	*Lymnaea stagnalis*	196	2.55	5	DRLF1; DRLF3; DRLF4; DRXI2; DRXI3
	*Radix* sp.	104	2.9	3	DRLFO1; DREC1; DREC2
National Domain of Chambord (DNC)	*Planorbis* sp.	1771	2.15	38	DCLF37; DCLF39–DCLF42; DCLF44;DCLF45;DCLF48; DCLF53;DCLF54
					DCLF57; DCLF59; DCLF61; DCLF64;DCLF68; DCLF71; DCLF72; DCLF75; DCLF76
					DCLF78; DCLF79; DCLF80; DCLF82; DCLF86; DCLF92 to DCLF103; ECDC16; ECDC26
	*Stagnicola* sp.	278	1.8	5	DCLF43; DCLF88; DCLF89; DCLF90; DCLF91
	*Anisus* sp.	144	2.08	3	DCLF73; DCLF74; DCL77
Zebulle Park Chevenon (ZE)	*Radix* sp.	248	1.2	3	ZELF1; ZELF2; ZELF3
Jouaignes (JO)	*Stagnicola* sp.	45	11.1	5	JOLF1; JOLF2; JOEC1. JOEC2; JOEC3

According to the morphological type of emitted cercariae and their origin, some snails were used to evaluate identification by the MALDI-TOF approach versus characterization by molecular biology: one *Radix* was positive with ocellated pigmented furcocercariae (FO), three with furcocercariae with or without eye spots (LF), and two with cercariae of Echinostomatidae (EC); five *Lymnaea stagnalis,* three positive with LF, two with xiphidiocercariae (XI); 10 *Stagnicola* sp., eight positive with LF and two with EC; three *Anisus* sp. with LF; 38 *Planorbis* sp., 36 with LF, and two with Echinostomatidae (Table 2).

**Table 2:** Molecular identification of processed cercariae.

Cercarial reference	Cercarial identification	Genbank accession numbers obtained in the present study (in red) and homologies with those extracted from Genbank (in black). In grey, no molecular identification.		
		D2	ITS2	ITS1
FODR1	*Trichobilharzia anseri*	MK168701 100% with FJ93861	MK168683 100% with FJ469785	MK168665 99% with FJ469785
LFDR1	*Diplostomum pseudospathaceum*	MK168711 99% with KR269766	MK168684 100% with KR269766	MK168668 99% with KR269766
LFDR3		MK168712 99% with KR269766	MK168685 100% with KR269766	MK168669 99% with KR269766
LFDR4		MK168713 99% with KR269766	MK168686 100% with KR269766	MK168670 99% with KR269766
LFZE1	*Tylodelphys* sp.	MK168714 no homology	MK168689 99% with KY462834	MK168673 99% with *Tylodelphys clavata* JQ665459
LFZE2		MK168715 no homology	MK168690 99% with KY462834	MK168674 99% with *Tylodelphys clavata* JQ665459
LFZE3		MK168716 no homology	MK168691 99% with KY462834	MK168675 99% with *Tylodelphys clavata* JQ665459
LFJO1	*Australapatemon* sp.	MK168709 99% with *Australapatemon burti* MF398342	MK168687 98% with *Australapatemon burti* JX9777785	MK168671 99% with KY570947
LFJO2		MK168710 99% with *Australapatemon burti* MF398342	MK168688 98% with *Australapatemon burti* JX9777785	MK168672 99% with KY570946
LFDC42		MK168703 99% with *Australapatemon burti* MF398342		MK168666 99% with KY570947
LFDC48		MK168724 99% with *Australapatemon burti* MF398342		MK168667 99% with KY570947
LFDC86		MK168735 99% with *Australapatemon burti* MF398342		MK500246 99% with KY570947
LFDC43	*Cotylurus* sp.	MK168704 99% with *Cotylurus cornutus* KY513182		
LFDC88		MK168726 99% with *Cotylurus cornutus* KY513182		
LFDC89		MK168736 99% with *Cotylurus cornutus* KY51318		
LFDC90		MK168738 99% with *Cotylurus cornutus* KY513182		
LFDC91		MK168739 99% with *Cotylurus cornutus* KY513182		
LFDC83	*Posthodiplostomum* sp.	MK168734 no homology		MK500245 99% with *Posthodiplostomum cuticola* MF171001
LFDC96	*Parastrigea* sp.	MK168740 98% with *Apharyngostrigea cornu* MF398345		MK500247 99% with *Parastrigea robusta* MF537208
LFDC37	*Alaria alata*		MK168699 100% with JF340222	
LFDC39		MK168745 100% with AF184263	MK168700 100% with JF340222	
LFDC40		MK168746 100% with AF184263		
LFDC41		MK168702 100% with AF184263		
LFDC44		MK168705 100% with AF184263		
LFDC45		MK168706 100% with AF184263		
LFDC53		MK168707 100% with AF184263		
LFDC54		MK168708 100% with AF184263		
LFDC57				
LFDC59				
LFDC61				
LFDC64		MK168729 100% with JF340217		
LFDC68		MK168737 100% with JF340217		
LFDC71				
LFDC72		MK168725 100% with JF340217		
LFDC73		MK168728 100% with JF340217		
LFDC74				
LFDC75				
LFDC76				
LFDC77				
LFDC78				
LFDC80				
LFDC82				
LFDC92				
LFDC93				
LFDC94				
LFDC95				







LFDC97		MK168741 100% with JF340217		
LFDC98		MK168742 100% with JF340217		
LFDC99		MK168743 100% with JF340217		
LFDC100		MK168730 100% with JF340217		
LFDC101		MK168731 100% with JF340217		
LFDC102		MK168732 100% with JF340217		
LFDC103		MK168733 100% with JF340217		
ECDC16	*Echinoparyphium* sp	MK168717 99% with *Echinoparyphium ellisi* KY436410		
ECDC26		MK168744 99% with *Echinoparyphium ellisi* KY436410		
ECJO1	*Echinostoma revolutum*	MK168720 100% with KP065596	MK168694 99% with AY68930	MK168678 99% with KM20150
ECJO2		MK168727 100% with KP065596	MK168695 99% with AY68930	MK168679 99% with KM20150
ECJO3		MK168721 100% with KP065596	MK168696 99% with AY68930	MK168680 99% with KM20150
ECDR1	*Petasiger phalacrocoracis*	MK168718 100% with JQ425593	MK168692 100% with JQ425593	MK168676 99% with KJ720683
ECDR2		MK168719 100% with JQ425593	MK168693 100% with JQ425593	MK168677 99% with KJ720683
XIDR2	*Plagiorchis* sp	MK168722 99% with KF533392	MK168697 no homology	MK168681 99% with JX522536
XIDR3		MK168723 99% with KF533392	MK168698 no homology	MK168682 100% with JX522536

FO: ocellated pigmented furcocercariae, LF: furcocercariae with or not eyes spots, EC: cercariae of Echinostomidae, XI: Xiphidiocercariae, DR: Der-Chantecocq lake, DC: National Domain of Chambord, ZE: Zebulle Park Chevenon, JO, Jouaignes.

Twelve taxa were identified according to the domains used (D2, ITS1 et ITS2) and after comparison of their percentage of homology with sequences deposited in genbank: FO: *Trichobilharzia anseri*; LF: *Diplostomum pseudospathaceum, Tylodelphys* sp*., Australapatemon* sp., *Cotyluru*s sp., *Posthodiplostomum* sp., *Parastrigea* sp., *Alaria alata;* EC: *Echinostoma revolutum*, *Petasiger phalacrocoracis*, *Echinoparyphium* sp.; XI (*Plagiorchis* sp.) (Table 3).

**Table 3:** Molecular identification of snail hosts.

Reference snails	Identification of snails	Genbank accession numbers obtained in the present study (in red) and homologies with those extracted from Genbank (in black). In grey, no molecular identification.	
		16S	Cox1
DRFO1	*Radix* sp.		
DRLF1	*Lymnea stagnalis*		
DRLF3	*Lymnea stagnalis*		
DRLF4	*Lymnea stagnalis*		
ZELF1	*Radix auricularia*	KM168747 99% with KP098540	MK124575 no homology
ZELF2	*Radix* sp.		
ZELF3	*Radix auricularia*	KM168747 99% with KP098540	
JOLF1	*Stagnicola palustris*	KM168749 99% with HQ659900	MK124565 no homology
JOLF2	*Stagnicola palustris*	KM168750 99% with HQ659900	MK124566 no homology
DCLF42	*Planorbis planorbis*		MK124571 99% with JQ776562
DCLF48	*Planorbis* sp.		
DCLF86	*Planorbis planorbis*	KM168751 99% with JQ776562	
DCLF43	*Stagnicola palustris*	KM168752 99% with HQ659900	MK124567 no homology
DCLF88	*Stagnicola palustris*	KM168753 99% with HQ659900	MK124568 no homology
DCLF89	*Stagnicola palustris*	KM168754 99% with HQ659900	MK124569 no homology
DCLF90	*Stagnicola* sp.		
DCLF91	*Stagnicola* palustris	KM168755 99% with HQ659900	MK124570 no homology
DCLF83	*Planorbis planorbis*	KM168756 99% with JQ776589	MK124573 99% with JQ776562
DCLF96	*Planorbis planorbis*	KM168757 99% with JQ776589	
DCLF37	*Planorbis* sp.		
DCLF39	*Planorbis* sp.		
DCLF40	*Planorbis* sp.		
DCLF41	*Planorbis* sp.	KM168758 97% with JQ776589	
DCLF44	*Planorbis* sp.		
DCLF45	*Planorbis planorbis*	KM168759 99% with JQ776588	
DCLF53	*Planorbis* sp.		
DCLF54	*Planorbis planorbis*	KM168760 99% with JQ776588	
DCLF57	*Planorbis planorbis*	KM168761 99% with JQ776588	
DCLF59	*Planorbis planorbis*	KM168762 99% with JQ776588	
DCLF61	*Planorbis planorbis*	KM168763 99% with JQ776588	
DCLF64	*Planorbis planorbis*	KM168764 99% with JQ776588	
DCLF68	*Planorbis* sp.		
DCLF71	*Planorbis* sp.		
DCLF72	*Planorbis planorbis*	KM168765 99% with JQ776587	MK124572 99% with JQ776562
DCLF73	*Anisus* sp.	KM168766 no homology	
DCLF74	*Anisus* sp.	KM168767 98% with AY577477	
DCLF75	*Planorbis planorbis*	KM168768 99% with JQ776588	
DCLF76	*Planorbis* sp.		
DCLF77	*Anisus* sp.	KM168769 no homology	
DCLF78	*Planorbis planorbis*	KM168770 99% with JQ776587	
DCLF80	*Planorbis* sp.	KM168771 98% with JQ776589	
DCLF82	*Planorbis planorbis*	KM168772 99% with AY577476	
DCLF92	*Planorbis planorbis*	KM168773 99% with JQ776587	
DCLF93	*Planorbis* sp.		
DCLF94	*Planorbis* sp.		
DCLF95	*Planorbis* sp.		
DCLF97	*Planorbis* sp.		
DCLF98	*Planorbis* sp.		
DCLF99	*Planorbis* sp.		
DCLF100	*Planorbis* sp.		
DCLF101	*Planorbis* sp.		
DCLF102	*Planorbis* sp.		
DCLF103	*Planorbis planorbis*	KM168774 99% with JQ776587	
DCEC16	*Planorbis planorbis*	KM168775 99% with JQ776589	
DCEC26	*Planorbis planorbis*	KM168776 99% with JQ776587	
JOEC1	*Stagnicola* sp.		
JOEC2	*Stagnicola palustris*	KM168777 99% with HQ659900	
JOEC3	*Stagnicola palustris*	KM168778 99% with HQ659900	
DREC1	*Radix balthica*	KM168779 99% with KP098541	MK124574 no homology
DREC2	*Radix* sp.		
DRXI2	*Lymnea stagnalis*		
DRXI3	*Lymnea stagnalis*		

FO: ocellated pigmented furcocercariae, LF: furcocercariae with or not eyes spots, EC: cercariae of Echinostomidae, XI: Xiphidiocercariae, DR: Der-Chantecocq lake, DC: National Domain of Chambord, ZE: Zebulle Park Chevenon, JO: Jouaignes.

Furcocercariae identified as *Cotylurus* sp. were detected in *Stagnicola palustris* from different closed ponds of the National Domain of Chambord. *Alaria alata* can use one of two intermediate hosts collected the same day in the same ponds: *Planorbis planorbis* (e.g., DCLF57/59/61/64) or *Anisus* sp. (DCLF73/DCLF74/DCLF77). Furcocercariae of *Australapatemon* sp. were observed in two species of snails from two distant sites (LFJO1/LFJO2 from *Stagnicola palustris* and LFDC42/LFDC48/LFDCLFDC84/LFDC86 from *Planorbis planorbis*).

**Figure 1:**
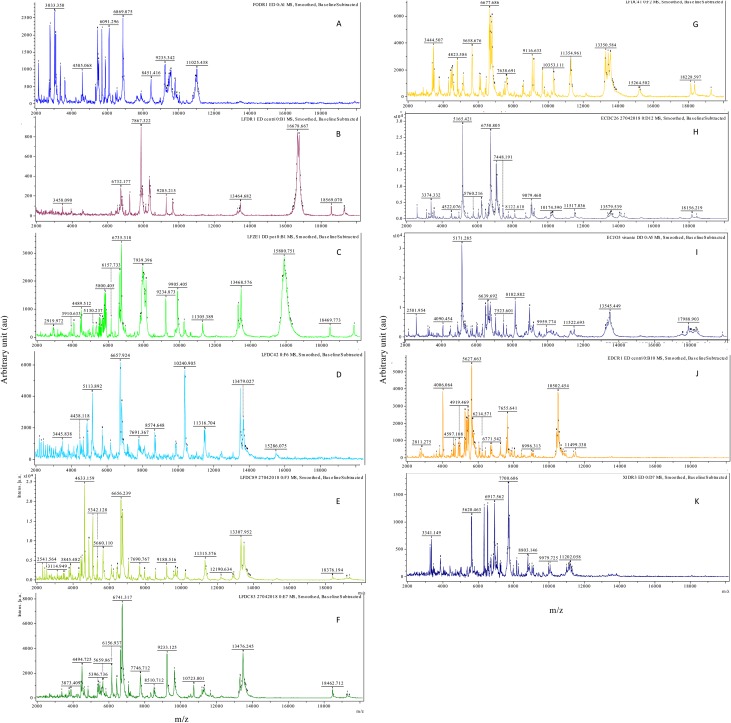
Representative MALDI-TOF spectra of cercariae obtained using a simple direct-deposit protocol. Flex Analysis software, smoothed spectra with baseline substracted A: *Trichobilharzia anseri* (FODR1) B: *Diplostomum pseudospathaceum* (LFDR1) C: *Tylodelphys* sp. (LFZE1) D: *Australapatemon* sp. (LFDC42) E: *Cotylurus* sp. (LFDC89) F: *Posthodiplostomum* sp. (LFDC83) G: *Alaria alata* (LFDC 41) H: *Echinoparyphium* sp. (ECDC26) I: *Echinostoma revolutum* (ECJO3) J: *Petasiger phalacrocoracis* (ECDR1) K: *Plagiorchis* sp. (XIDR3)

Consistent and reproducible MALDI-TOF MS spectra were acquired from all the specimens, except for LFDC96 (*Parastrigea* sp.) with peaks of high intensity between 2 and 20 kDa. All the 12 taxa from which spectra were acquired displayed different peak patterns (Fig. 1). Spectra were tested against the Bruker Taxonomy MSP library, providing no bacterial or fungal identification with log-score values > 1.5.

**Figure 2:**
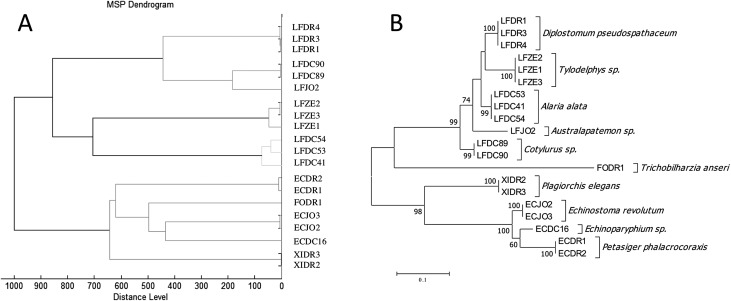
Panel A: Hierarchical clustering dendrogram of MALDI-TOF MSP, using the correlation distance measure and Ward algorithm. All the MSPs were included in the final database. Panel B: Maximum likelihood tree of the D2 domain of the cercariae strains present in the MALDI-TOF spectral database.

The results of MSP cluster analysis are shown in Figure 2A. Specimens of the same species were grouped together in clusters clearly separated from other species, with low intra-species heterogeneity. Furcocercariae with forked tails without pigmented eye spots were particularly distant from the other cercariae. The classification is consistent when compared to that based on molecular data inferred using the maximum likelihood method (Fig. 2B).

**Figure 3:**
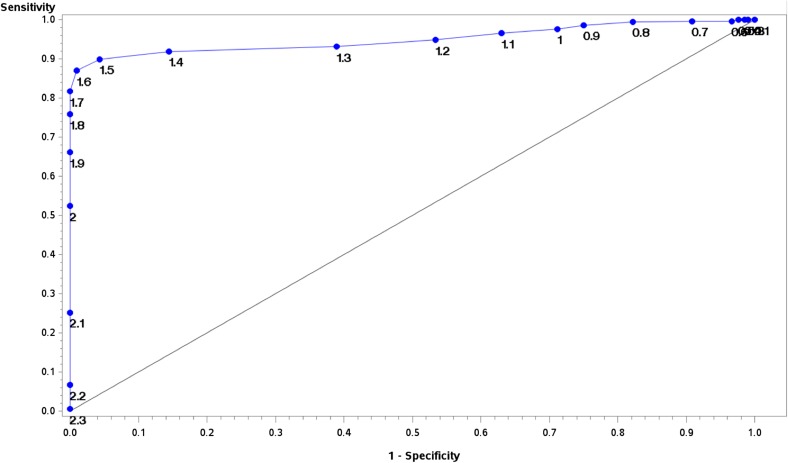
ROC curve for determination of the optimal LSV cut-off. Logistic regression model based on identification prediction using the final version of the MALDI-TOF cercariae database compared to molecular identification.

The MSP database constructed with 10 species (20 MSP) was blind-tested against spectra acquired from 22 samples of freshly emitted cercariae representing five species. Among them, three species were present in the database (*Alaria alata n* = 15/22, *Australapatemon* sp. *n* = 1/22, and *Echinoparyphium* sp. *n* = 1/22). Among the 1056 acquisitions, 264 spectra (25%) were flat-line spectra and were therefore not included in the analysis. In the 792 remaining spectra, 648 were acquired from species present in the database. Among them, only 147/648 (22.68%) reached the Bruker recommended cut-off LSV of 2.0 for species level identification. However, an LSV of 1.7 sufficient for genus identification was obtained in 443/648 spectra (68.36%). In order to evaluate the best LSV cut-off for cercariae identification, a logistic regression model, based on concordance of MALDI-TOF and molecular data was then built. The ROC curve is shown in Figure 3. The area under the curve of the model was 0.9501 (95% Wald confidence limits: 0.9357–0.9644). Choosing an LSV threshold of 1.7 enabled us to obtain specificity of 100%, with sensitivity of 81.7% (Fig. 3). No false identification was reported using 1.7 and 2.0 cut-off LSV, even for the taxa which were not present in the database, 108 spectra of *Cotylurus* sp. (LFDC43, LFDC88, LFDC89, LFDC90, LFDC91) and 36 spectra of the *Posthodiplostomum* sp. (LFDC83). The database was then updated to include *Cotylurus* sp. with MSPs generated from LFDC89 and LFDC90. This new version yielded good performance for species identification of *Cotylurus* sp. (78 correct identification among the 78/108 spectra attaining the LSV cut-off of 1.7). This updated database had similar performances with 521/792 spectra reaching the 1.7 cut-off (65.78%), with 100% correct identifications.

Retrospectively, we did not observe any differences in terms of spectral profile between cercariae of the same species from one year to the next. For example, spectra of *Alaria alata* isolated at Chambord (LFDC41/44/45/50/51/52/53/54) in 2017 did not differ from those isolated in 2018 (LFDC57–LFDC72). We also did not observe any difference between cercariae of the same species isolated from different sites: for example, *Australapatemon* sp. spectra from specimen LFJO1–LFJO2 isolated in the JO site were not different from those isolated from Chambord (LFDC42, LFDC48, LFDC84, LFDC86).

We observed in one case, re-emission of the same cercariae (LFDC88: *Cotylurus* sp.) by the same mollusc (DCLF88) 90 days later. There was no difference in the spectral profile between the first and the second emission.

In order to assess the potential use of this new tool, we evaluated the effect of ethanol conservation on analytical performance. We compared the LSV and the rate of true positives between freshly emitted cercariae in the blind validation specimens and cercariae of the same emission preserved over 3 months in 80% ethanol. Among the 1114 spectra acquired, 553 (49.64%) were flatline spectra. In the 561 spectra analyzed using the updated database, 183 reached the cut-off LSV (32.62% vs 65.78% without ethanol *p* < 0.0001). The mean LSV was 1.80 (Median: 1.93; Min: 0.13; Max: 2.77) for freshly emitted cercariae versus 1.62 (Median: 1.71; Min: 0.43; Max: 2.48; *p* < 0.0001) for cercariae preserved in ethanol. Interestingly, there was no false identification for the 183 spectra preserved in ethanol attaining the 1.7 cut-off.

**Figure 4:**
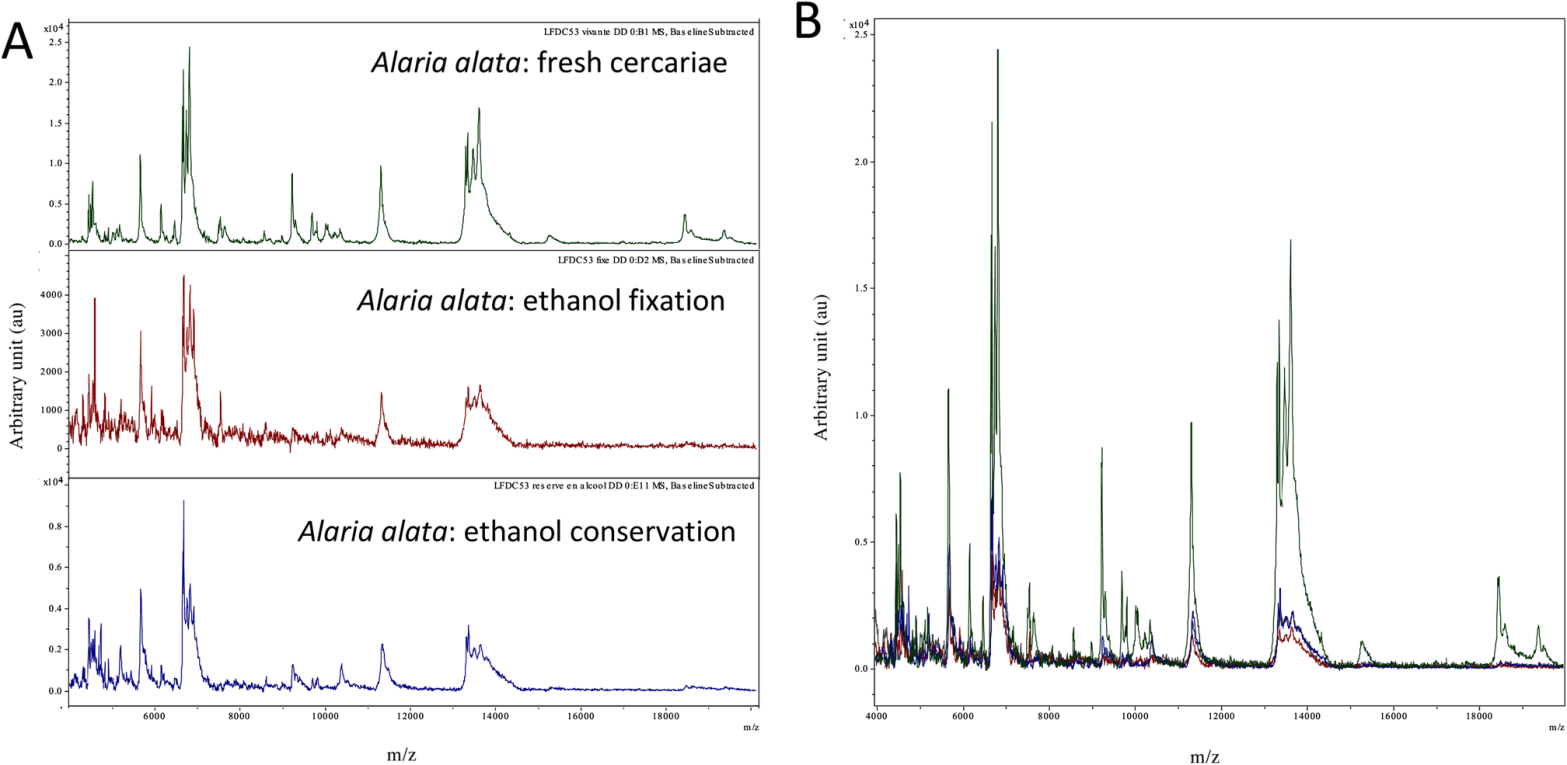
Panel A: Representative MALDI-TOF spectra of the same specimen (LFDC53) under the following conditions: fresh cercariae, ethanol fixation, and ethanol conservation (12 months). Panel B: Superposition of panel A spectra.

In a second round, we analyzed the effect of exposure time to ethanol on collected samples and freshly emitted samples. Among the 1074 spectra (including the 561 previously described), 287 (26.72%) reached the 1.7 LSV cut-off (all of them were concordant with molecular identification). There was no clear tendency of LSV to decrease as a function of preservation time. When comparing spectra obtained from fresh cercariae versus those obtained with cercariae fixed or conserved in ethanol, we mainly observed a degradation of peak intensity resulting in a lower signal-to-noise ratio. Representative spectra are shown in Figure 4.

## Discussion

We propose MALDI-TOF MS as a rapid and reliable identification system for cercariae.

This approach is easier to implement than morphological identification. Indeed, according to Gaillot et al. [13], identification of morphological differences between species of Trematoda at the cercaria stage relies on structures that can only be found in fresh cercariae (after contact with urine for the excretory system or carmine-borax staining). For cercariae with forked tails and with colorless eye spots, the morphological feature used is the position of the penetration glands: preacetabular (*Tylodelphys* sp., *Cotylurus* sp.) or postacetabular (*Diplostomum* sp. and *Australapatemon* sp.). For the last two taxa, the size of the glands and the body spinose are the morphological features used for diagnosis when emitted by *Lymnaea stagnalis* [10]. With regard to the cercaria emitted by planorbid snails [11], as for example those of *Alaria alata*, *Parastrigea* sp., and *Australapatemon* sp., other morphological features (number of rows of spines, size of spines around suckers, flame-cell formula and body spinose or not) are used to distinguish these genera. To avoid the use of several identification keys, it would be beneficial to have only one approach to the cercariae, regardless of the snail and its living environment.

Identification by molecular biology remains an expensive technique that requires trained staff as well as expertise in processing and interpreting the results.

In our study, we demonstrated the ability of MALDI-TOF MS to reliably identify cercariae using a simple protocol. This direct deposit protocol is particularly time-saving compared to morphological and molecular methods. It allows high-throughput identification with more than one hundred specimens processed per day.

MALDI-TOF MS technology is nowadays increasingly accessible to clinical and research laboratories. This approach is also cost-effective as only a small number of reagents are needed. The cost of identifying bacteria using a direct deposit protocol on reusable targets was evaluated at €0.12 per well [13].

We found good discriminatory power when differentiating between the studied groups. This encouraging analytical performance needs to be confirmed on a larger number of taxa, including closely related species such as *Diplostomum pseudospathaceum*, *D. spathaceum* and *D. phoxini*.

According to Bruker’s recommendations, LSVs under 1.7 were considered invalid identification. LSVs between 1.7 and 2.0 were considered valid at the genus level, and LSVs higher than 2.0 were considered reliable identification at the species level. In our study, using an LSV cut-off of 2.0 for identification at the species level was highly specific, but resulted in a high proportion of unidentified spectra. Lowering the cut-off to 1.7 allowed for the identification of a higher number of specimens with similar specificity. This cut-off value has already been proposed for species-level identification of filamentous fungi [4, 29]. Further studies are needed for the validation of this cut-off on upgraded spectral databases with a higher number of taxa.

In our study, we observed a high proportion of “flat-line” and low-quality spectra. These can be explained by the heterogeneity of the cercariae deposited in the MALDI-TOF target. In our experience, four deposits per sample is a good compromise between deposit and acquisition time, and generally enables identification of the sample with at least an LSV > 1.7 on one well.

We did not note any influence of the species of emitting mollusc. This allowed us to confirm the circulation of *Alaria alata* in *Planorbis* as well as in *Anisus*. We also have found the same species, *Australapatemon* sp., in two different locations on two different species of snails. There was also no spectral difference in the same species at different times of study, or between two emissions of the same cercarial species by a same mollusc. These results appear to show that the signal measured by MALDI-TOF mass spectrometry is specific to the studied cercariae and not artefacts of the mollusc or the living environment. MALDI-TOF MS therefore seems to be a reproducible method for cercariae identification. In this study, we observed emission of only a single type of cercariae by each positive snail. Co-infection with two trematodes in the same snail is rarely observed in natural conditions and usually concerns two morphotypes of associated cercariae (e.g., forked tail/Echinostomatidae; Echinostomatidae/xiphidiocercariae; furcocercariae with eye spots/xiphidiocercariae), as shown in experimental conditions on competitive antagonism [12, 25]. Even though two cercariae can be emitted at the same time by a single snail, no cases of associations with the same morphotype of cercariae have been reported.

Fixation of cercariae and their storage in ethanol leads to degradation of spectral intensity, resulting in a high proportion of unidentified spectra. This raises a problem for the retrospective study of collections stored in ethanol. The study of other storage methods for the biological material, such as freezing at different temperatures and other fixatives, seems important for the development of this technique.

We constituted for this study an MSP database with a limited number of Trematoda species. It must be improved by inclusion of new species to cover the broad range of Trematoda involved in veterinary or human medicine. The database would also be improved by increasing the number of strains for a given taxa [30].

Our study highlights the huge potential of MALDI-TOF for large epidemiological surveys of Trematoda.

This technique could thus be applied to the study of human schistosomiasis, including the detection of hybrids [3, 8, 24], allowing for rapid and precise identification of the cercariae obtained during large snail collection campaigns. It would be of particular interest in areas of mixed circulation. Another field of application is the environmental survey of flukes of interest in human and veterinary medicine.

## Conclusion

MALDI-TOF MS is a promising technique for cercariae identification at the species level. It has great discriminatory power using a rapid and easy preparation protocol. The implementation of a spectral database, gathering a large number of species, is one of our objectives for use in routine identification.
